# Policy options for mitigating impacts of COVID-19 on domestic rice value chains and food security in West Africa

**DOI:** 10.1016/j.gfs.2020.100405

**Published:** 2020-09

**Authors:** Aminou Arouna, Guillaume Soullier, Patricio Mendez del Villar, Matty Demont

**Affiliations:** a Africa Rice Center (AfricaRice), 01 BP 2551, Bouake 01, Cote d’Ivoire; b CIRAD, UMR ART-DEV, F-34398, Montpellier, France; c ART-DEV, Univ Montpellier, CIRAD, CNRS, Univ Montpellier 3, Univ Perpignan Via Domitia, Montpellier, France; d CIRAD, UMR TETIS, F-34398, Montpellier, France; e TETIS, Univ Montpellier, AgroParisTech, CIRAD, CNRS, INRAE, Montpellier, France; f International Rice Research Institute (IRRI), Los Baños, Laguna, Philippines

**Keywords:** Food security, Food policy, Foodborne zoonotic pathogens, Trade disruption, Impact, West Africa

## Abstract

Rice plays a strategic role in food security in West Africa. However, the region increasingly relies on rice imports due to a growing and structural deficit, and domestic value chains face constraints in technology, finance and coordination. As a result, West Africa is very vulnerable to international and local trade disruptions, such as the ones currently inflicted by the COVID-19 pandemic. We build on evidence of the current state of domestic rice value chain upgrading in West Africa to anticipate the impacts of the COVID-19 pandemic on rice value chains’ resilience and their capacity to sustain food security in the region. Several policy options are proposed to help West African governments mitigate the impacts of the COVID-19 crisis on food security.

## Highlights

• Rice plays a strategic role in food security in West Africa.
• Growing reliance on rice imports exposes the region to international trade disruptions.
• We assess the resilience of domestic rice value chains to COVID-19.
• We anticipate the potential impact of foodborne zoonotic pathogens on food security.
• We propose policy options for mitigating impacts of the pandemic in West Africa.

## 1 Context

**Fig. 1:**
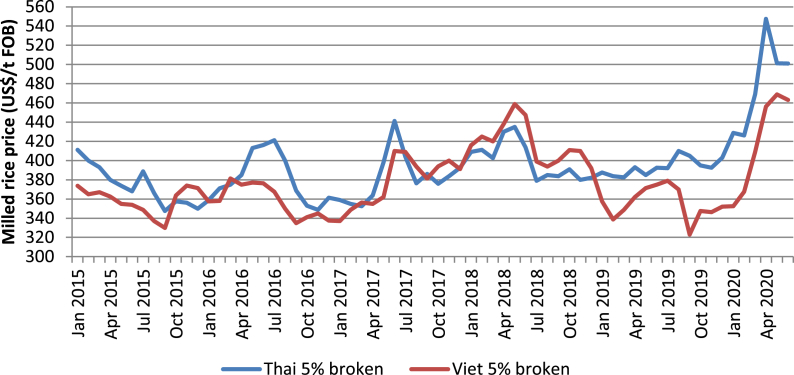
Evolution of international rice prices on the world market, January 2015–June 2020. Notes: FOB = Free On Board (FOB). Thai and Viet refer to rice imports from Thailand and Vietnam, respectively. Source: OSIRIZ/InfoArroz (2020).

Food insecurity remains prevalent in West Africa. During 2009–2018, the number of undernourished people in the region almost doubled from 32 to 56 million or 15% of the West African population, while globally, it decreased from 842 to 822 million (FAO et al., 2019). Rice increasingly plays a strategic role in food security in West Africa, where annual per capita consumption levels rose five-fold in the last six decades and are currently the highest on the continent. Production increased during the same period (USDA, 2019), but as a result of rapid demographic growth (2.7% annually) and diet changes, the region increasingly relies on rice imports (Mendez del Villar and Lançon, 2015). This renders West Africa very vulnerable to international trade disruptions such as the ones currently inflicted by the corona virus disease (COVID-19) crisis. A prolonged pandemic can cause price increases due to disruptions in distribution chains and trade flows. World rice prices have been continuously increasing over the 12-month period March 2019–March 2020, featuring a steep upward sloping trend since the outbreak of the COVID-19 pandemic in December 2019 (Fig. 1). In May 2020, this upward slope was interrupted for the first time, but it is uncertain at this point how rice prices will evolve from here onwards as a second wave of the pandemic is not excluded.

The increase in rice imports in West Africa is partly due to the low quality of locally-produced rice which is largely supplied by fragmented, traditional value chains with little coordination between farmers, millers and traders. Sourcing paddy is mostly done through spot market transactions with little quality differentiation. As a result, domestic rice is often an inferior substitute for imports and domestic and global rice markets are poorly integrated (Demont, 2013). Apart from higher quality standards and lower variability and heterogeneity in rice quality, import value chains have other competitive advantages such as their superior dynamism and capitalization, thanks to better access to finance (Mendez del Villar and Lançon, 2015). Consequently, when rice prices spike on the world market, domestic rice value chains fail to rapidly respond and compete against import value chains.

Framed field experiments have revealed that local rice struggles to compete with imports even if its quality is upgraded to import standards (Demont et al., 2017). To meet these quality standards and satisfy urban consumers, rice value chains require substantial investment in modernization through process, product, functional (e.g., vertical coordination such as contract farming or vertical integration) and channel upgrading (e.g., expanding domestic value chains into import-biased urban markets) (Demont, 2013). Integration of domestic rice in import channels (wholesale and retail) is however challenging (Mendez del Villar and Lançon, 2015), as it may require close coordination among multiple stakeholders such as importers, retailers, wholesalers, millers and farmers.

Soullier et al. (2020) recently compiled evidence indicating that upgrading of domestic rice value chains has progressed in West Africa in the decade following the 2008 food crisis. In 2019, 57 modern mills were operating in the region, some of them sourcing paddy directly from farmers through contract farming, and others directly managing production of rice through vertical integration. Depending on the progress in the modernization of their rice value chains, West African countries were classified into three groups:

• Group 1 includes the countries with the highest rice import bills and paddy production, and where the modernization of rice value chains is most advanced, i.e. Nigeria and Senegal.
• Group 2 features countries with lower rice import bills and paddy production, and where the modernization of rice value chains is slowly emerging, i.e. Ghana, Mali, Côte d’Ivoire, Burkina Faso, Liberia, Niger, Sierra Leone, Benin and Togo.
• Group 3 is composed of countries where little investment in rice value chain upgrading is observed, i.e. Guinea, Mauritania, The Gambia and Guinea-Bissau.

The evidence also suggests that most farmers remain connected to final rice markets through traders and small and medium scale millers. Indeed, among the estimated four million rice growers in West Africa in 2019, 99.74% marketed paddy through spot or interlinked transactions without any formal coordination between millers and farmers (Soullier et al., 2020). Traditional value chains therefore remain the core providers of food security in West Africa.

The evidence enables anticipating the effects of foodborne zoonotic pathogens on domestic rice value chains' resilience and capacity to sustain food security in West Africa. COVID-19 has emerged in December 2019 in China and in a period of three months, about 188 countries around the world were contaminated, including every single country in Africa. To slow down the speed of contamination, movement restrictions, curfews and complete lockdowns were imposed in many parts of the world. The economic impact is already visible after three months of the pandemic; economic growth in Sub-Saharan Africa is projected to tumble from +2.4% in 2019 to between –2.1 and –5.1% in 2020, announcing the region's first recession in more than two decades (Calderon et al., 2020). The COVID-19 pandemic may further create disruptions in domestic rice value chains, and exacerbate West Africa's dependency on rice imports. On the other hand, the pandemic could also offer an opportunity to domestic rice value chains if African states are forced to limit imports due to a fall in foreign exchange linked to the reduction in exports of agricultural and mining products. The purpose of this Perspectives Article is to discuss the potential impacts of the COVID-19 pandemic on domestic rice value chains' resilience and their capacity to sustain food security in the region, in order to devise policy measures that can successfully mitigate this impact. Apart from producers, we will also focus on the mid-stream section (millers and traders), in particular, as this so-called “hidden middle” fulfills a crucial intermediary role between production and consumption, which is essential in sustaining rice value chains' capacity of providing food security in the region (Soullier and Moustier, 2020).

## 2 Potential impacts of COVID-19 on rice value chains

With the increasing spread of COVID-19 and different restriction measures, both traditional and upgraded domestic value chains are likely to be affected in the short, medium and long term. To assess the potential impact of the COVID-19 pandemic on domestic rice value chains' resilience and their capacity to sustain food security in the region, we apply Porter (1985) business model assessment on six fundamental value chain operations:

1. *Procurement.* The spread of COVID-19 may affect the procurement of paddy for traditional and upgraded mills. Countries in Group 2 and 3 dominated by traditional mills are more likely to experience disruptions in the procurement of paddy as a result of reductions in mobility of informal traders caused by lockdowns imposed by governments in response to COVID-19. In contrast, modern mills that directly coordinate with farmers through contract farming (vertical coordination) or directly organize own rice growing (vertical integration) would depend less on informal trade. Soullier et al. (2020) found that the supply of paddy is the most important driver of private investment in modern milling infrastructure. However, the adoption of contract farming by mills in West African rice value chains is only marginal, so we expect modern mills to be affected by supply disruptions as well. Furthermore, the crisis creates other disruptions in modern rice value chains, e.g., by providing incentives for contracted farmers to side-sell paddy. As price volatility is high in the crisis period, farmers may default on contracts because differences between contract and spot market prices may be even greater than in periods of relative stability. In such crisis context, relational and geographical proximity is often the most efficient institution to enforce contracts. Furthermore, if the pandemic persists, local rice production may decrease due to labor shortage, input shortage and costs, high death rate due to disease, etc. This might lead to procurement issues for both traditional and upgraded mills in all countries. Alternatively, it is also possible that in the midterm, under high world prices, producers respond by increasing area and input use (if input channels are not disturbed), expecting that this price trend will persist. This may increase the quantity of paddy that traditional and upgraded mills can procure and relieve pressure on prices. This will negatively affect producers, because prices will decrease in the midterm, but it may increase millers' margins. However, inflated prices are likely to be short-lived. Since May 2020, there were some early signs of price stabilization or even a downward trend thanks to Vietnam's and India's reopening of their rice export markets and a revival of competition between the main Asian exporters (Fig. 1). In the coming months when new crops from Asia will begin to arrive on export markets, prices are expected to fall due to oversupply of rice and a possible decline of world demand due to decrease of purchasing power of consumers. This suggests that a possible 2020 rice crisis will likely be caused by a drop in demand, rather than supply (which was the main factor during the 2007–2008 crisis).
2. *Logistics.* In many West African countries, governments have allowed free movement within the country of “essential goods” such as food, contrarily to human movement. Therefore, both inbound and outbound logistics should not be significantly affected by the current COVID-19 spread. In fact, in Nigeria we observed massive movement of rice seedlings from Cross River State all the way to Kano (i.e. about 911 km), despite the COVID-19 inflicted restrictions on mobility. However, movement of goods is not completely independent from movement of people.
3. *Finance.* This is a major constraint hampering domestic rice value chain upgrading. Financing rice growing is a common constraint among family farmers. However, small-scale millers face similar constraints. They rarely have access to formal credit from banks and rely on their own savings. This is also the case for upgraded mills, particularly when they are domestic actors. The spread of COVID-19 has substantially increased uncertainty surrounding economic activities and therefore will increase financial institutions' reluctance to provide loans. Therefore, commercial financial institutions may reduce their credit lines. This will particularly affect upgraded mills, which require high amounts of credit to maintain operational funds to pre-finance rice producers and to collect sufficient volumes of paddy to fill their capacity and reach profitability.
4. *Processing.* The spread of COVID-19 will not alter processing activities in both traditional and upgraded mills. The use of face masks is already a recommended practice in rice mills. However, the application of social distancing can reduce labor productivity and efficiency to some extent, especially in large milling facilities. The major risk is that a single case of COVID-19 contaminated staff may lead to a complete lockdown of the facilities and temporary unemployment. On the other hand, traditional mills may gain from the drastic decrease in oil prices on the world market.
5. *Human resources and labor.* Due to restrictions of mobility and lockdown measures, milling facilities may experience shortage of labor. This may affect traditional mills to a lesser extent since they are usually operated by a single person or family. In the case of upgraded mills, workers may have travelled to other regions or may not have free mobility to travel from and to the mill. This may lead to severe labor shortages and significantly impact upgraded mills' operations, which rely more on specialized labor.
6. *Marketing and sales.* The massive loss of jobs resulting from lockdowns will reduce purchasing power of consumers, reduce demand and jeopardize food security of households. West Africa imports about 46% of its rice consumption mainly from Asia. With many rice export countries in Asia being heavily affected by COVID-19, further reductions of rice imports into West Africa are not excluded in the short term. To prioritize national food security, some rice exporters such as Vietnam, Cambodia and India implemented temporary export restrictions as of the beginning of April 2020. This resulted in a steep increase in world rice prices (Fig. 1) until May 2020, when Vietnam resumed exports.^1^ Whether rice prices will now increase again, stabilize at the current higher level or decline is uncertain and will largely depend on the upcoming actions of major players in the rice world market (e.g., China, India and Thailand). Panic buying due to uncertainty related to the pandemic may further contribute to price increases. In the short term, price increases of paddy and milled rice were already observed in Ghana, Cote d’Ivoire, Nigeria and Niger in March 2020. Price increases will benefit value chain actors such as producers, traders, traditional millers and upgraded mills. However, considering a global value chain approach, export supply chains are currently being disrupted due to logistical reasons, but not due to global rice shortages and world prices may not remain high. Indeed, when the next Asian harvest starts arriving in October–November 2020, export supply may be abundant and world prices may collapse, which means that for African countries, it will be more advantageous to import. For instance, early April 2020, China was encouraging producers to increase rice production in anticipation of a possible supply disruption in the coming months (Fei and Ni, 2020). An increase in export supply and decrease of world prices may negatively affect demand for local rice *vis-à-vis* imported rice and decrease sales of domestic rice.

The COVID-19 crisis will also affect farmers, which are the main suppliers of paddy in rice value chains. Below we summarize the effect of COVID-19 on farmers in terms of input access and output markets:

1. *Input access.* Whereas there are little or no imports of phosphorus rock/phosphoric acid for fertilizer in West Africa, most of the urea and potash needs for fertilizer are met by imports. With the lockdowns due the COVID-19 pandemic, production plans of fertilizer in exporting countries are disrupted or delayed. Movement of intermediate inputs including fertilizers and pesticides has slowed down. In addition, local input dealers may also face challenges in the procurement of fertilizers and insecticides. Therefore, there may be supply shortage of intermediate inputs, which may result in higher prices. In rice value chains, access to seeds may also be a challenge. Due to the crisis and low purchasing power, farmers may also respond by consuming their rice stocks, including seed. This was already observed during the recent Ebola crisis in West Africa. Rice production in West Africa is labor intensive and access to labor may also be a challenge due to lockdowns. Technology transfer and access to improved practices could slow down due to the inactivity of the national extension agencies and NGO staff compelled to terminate operations as a result of the spread of COVID-19. This will limit farmers' access to main intermediate inputs (fertilizer, pesticides, seeds and labor) and technical know-how and will negatively affect production. Moreover, similarly to millers, farmers' finance constraints may be further exacerbated by COVID-19.
2. *Output markets.* Under a lockdown, a collapse of local food supply systems is likely. Indeed, farmers may not be allowed to go to the market for selling their products and may not have access to alternative marketing strategies (e.g., digital marketing). Although rice paddy is less perishable compared to other food items, farmers' challenge to timely sell paddy may increase their liquidity constraints and jeopardize their food security.

## 3 Policy options

The extent of the impacts of the COVID-pandemic on rice value chains in West Africa will depend on numerous factors, including the severity and duration of the health impacts, the macroeconomic shocks and the effectiveness of decision makers' responses. Policy measures should not only adapt to the current emergency to avoid the health crisis “morphing into a food crisis” (Roy-Macauley et al., 2020), but also prepare food systems to the future “new normal”. To reduce the current and potential impact of the COVID-19 pandemic on domestic rice value chains’ resilience and their capacity to sustain food security in West Africa, we propose the following policy options in the short, medium and long term:

### 3.1 Short term policy options

• We recommend policy makers to provide financial support to the “hidden middle”, particularly rice millers, as crucial intermediaries in both traditional and upgraded rice value chains. Indeed, value chain finance targeting continuity of operations and improved coordination between millers and farmers could help addressing challenges both at farm and mill levels. Operational credit should be facilitated in all countries to avoid food shortage and value chain actors' deficits. In particular, governments of countries in Group 2 and 3 (Ghana, Mali, Côte d’Ivoire, Burkina Faso, Liberia, Niger, Sierra Leone, Benin, Togo, Guinea, Mauritania, The Gambia and Guinea-Bissau) are advised to provide financial support (e.g., interest-free loans) to both traditional and upgraded mills to ease the procurement of rice paddy during the pandemic period as we deem this the most crucial operation enabling rice value chains to sustain food security in these countries. In addition, governments should facilitate access to finance through the reduction of loan administrative protocols to reduce complexity of procedures during the pandemic. This financial support should be conditional to millers applying acceptable prices and margins to avoid speculation.
• With increasing partial unemployment and decreasing overall purchasing power, continuous engagement with millers is necessary to keep marketing margins at acceptable levels to avoid surges in local rice prices.
• Many West African countries have already allowed free movement of food (both paddy and milled rice) and we recommend this policy to be maintained, emphasized and monitored to foster efficient rice value operations during the pandemic period.
• Governments should avoid lockdowns of milling plants. Instead, they should impose strict sanitary measures in milling facilities and provide special clearance passes to mill staff to enable uninterrupted processing of rice.
• Governments can buy local, milled rice from millers and maintain rice stocks to provide social safety nets to poor and vulnerable populations. Alternatively, similarly to India, governments can buy paddy rice from farmers and request rice millers to custom mill fixed amounts of paddy at fixed rates, thereby also contributing to maintaining paddy flows and keeping mills operational. Safety nets could be extended to farmers whose subsistence is negatively affected by the lockdown.
• Governments can delay payment terms of millers' electricity bills during the pandemic to reduce mills' operational costs and maintain asset liquidity in their crucial role of intermediating between paddy suppliers (farmers) and consumers.
• With disruptions in the intermediate input market, governments should organize local production of fertilizers and subsidize key intermediate inputs such as quality seeds, fertilizers and pesticides to compensate farmers for productivity losses.

### 3.2 Medium and long term policy options

• Governments are recommended to provide a supporting enabling environment for crowding in domestic and foreign direct investment in rice value chain upgrading (Soullier et al., 2020); direct government investment should be avoided.
• Governments can develop special credit lines for investors in modernization of rice milling infrastructure such as warehouses, facilities, or feeder roads around their factories to enhance rural logistics and increase rice value chains' performance and resilience. Such credits should be made available in particular to small-scale millers to help them upgrade their technologies. They are indeed the ones who are expected to be able to collect the highest paddy volumes, because their collection network is already developed (Soullier and Moustier, 2020).
• Governments are recommended to develop a regulatory framework for contract farming as this can facilitate secured procurement of quality paddy by mid-stream actors (millers), reduce the number of intermediaries and reduce reliance on informal markets. It is expected that better vertical coordination between actors can render rice value chains more resilient to climate and health shocks (Vroegindewey and Hodbod, 2018). Countries in Group 1 (Nigeria and Senegal) represent good examples to be emulated (Soullier et al., 2020).
• Support should be provided for R&D and value addition on (i) the development of cost-efficient bioethanol production from rice-straw, which is carbon neutral and less competitive to food supplies, to reduce the energy cost for mill plants; (ii) the digitization and automation of rice value chains; (iii) food safety; and (iv) enhancing the quality of rice-based diets (e.g., through diversification) and improving the nutrient content and composition of rice (e.g., parboiled rice, low glycemic index rice, micro-nutrient (bio)fortified rice) to enhance the health of populations and improve their resilience against diseases such as COVID-19. Improving the nutritional and qualitative aspects of production and educating the consumers about healthier rice-based product options can further contribute to a sustainable milling sector through the creation of diversified market opportunities.
• Governments are advised to continue developing rural infrastructure, especially around mill plants, to reduce inbound and outbound logistics costs, as costs related to inbound logistics are often passed on to farmers or small traders.

## Source of funding

This research was funded by the CGIAR Program on Rice and the Bill & Melinda Gates Foundation, Seattle, WA, USA [Grant no. OPP1194925].

## Declaration of competing interest

The authors declare that they have no known competing financial interests or personal relationships that could have appeared to influence the work reported in this paper.

## Footnotes

1 https://en.vietnamplus.vn/pm-allows-rice-export-resumption-from-may-1/172510.vnp.
